# Partial Remission of Diabetes in a Young Adult While Testing Positive for Several Islet Cell Autoantibodies: A Case Report, Literature Review, and Patient Perspective

**DOI:** 10.7759/cureus.25746

**Published:** 2022-06-08

**Authors:** Samson O Oyibo

**Affiliations:** 1 Diabetes and Endocrinology, Peterborough City Hospital, Peterborough, GBR

**Keywords:** honeymoon phase, latent autoimmune diabetes, islet cell autoantibodies, ketosis prone diabetes, patient perspective, treatment-free remission, type 1 diabtes

## Abstract

Type 1 diabetes is a chronic disease characterized by abnormal metabolism and hyperglycemia due to insulin deficiency. There is a rapid decline in insulin production due to autoimmune destruction of the pancreatic beta cells. Partial remission (honeymoon phase) of type 1 diabetes is common in children and young adults with newly diagnosed type 1 diabetes. There is temporary restoration of beta cell function such that little or no exogenous insulin is required. Stopping insulin therapy soon after an emergency admission requiring intravenous insulin and subsequent subcutaneous insulin therapy can be frightening for both patient and healthcare provider. Affected patients require education and support during this period. This report describes a case of a 28-year-old man who presented to the emergency department with features of type 1 diabetes and diabetic ketoacidosis. He was treated with intravenous fluids and intravenous insulin and discharged on a subcutaneous insulin regimen. Despite testing positive for several types of islet cell autoantibodies, the patient was able to stop insulin therapy within three months of diagnosis. The patient maintained a self-initiated low-carbohydrate diet, regular weight-reducing exercise, and normal glucose levels without the need for insulin therapy. The honeymoon phase of type 1 diabetes, latent autoimmune diabetes, and ketosis-prone type 2 diabetes are discussed as important differential diagnoses.

## Introduction

Type 1 diabetes is a chronic disease characterized by abnormal metabolism and hyperglycemia due to a rapid decline in insulin production from the pancreatic beta cells. There is underlying autoimmune destruction of the pancreatic beta cells by islet cell autoantibodies. Patients generally present with polydipsia, polyuria, and weight loss. Insulin therapy is usually required at diagnosis and for maintenance. The main islet cell autoantibodies are glutamic acid decarboxylase autoantibodies (GADA), tyrosine phosphatase-related islet antigen-2 autoantibodies (IA-2A), insulin autoantibodies (IAA), zinc transporter 8 autoantibodies (ZnT8A), and non-specific islet cell cytoplasmic autoantibodies (ICA). The risk of developing type 1 diabetes is markedly increased when islet autoantibodies to two or more of the antigen groups are found in the general population [1].

The honeymoon period (temporary, partial remission) of type 1 diabetes mellitus is characterized by reduced insulin doses required to maintain good glycemic control. This can happen soon after diagnosis and could last months to years. There is transient restoration and preserved function of the existing beta cells on initiation of insulin therapy for a variable period such that patients require much less insulin or none at all, until there is complete beta cell dysfunction [2]. A few patients experience a prolonged honeymoon phase, where no insulin is required to maintain normal blood glucose levels. This period of insulin independence has become a window of opportunity for research into prolonging beta cell survival with immune modulation, with the aim of halting the progression to restarting insulin (insulin-dependence) [3].

Despite clinical remission of type 1 diabetes being a well-known and extensively researched phenomenon in children, little is known about the pathogenesis, management, and prognosis of this phenomenon in adults. This is a “watch and wait” period, during which time the patient can suddenly become insulin-dependent. Affected patients require continued monitoring, guidance, and reassurance during this period. This report describes a case of a young man who presented to the emergency department with biochemical features of type 1 diabetes with diabetic ketoacidosis. Despite testing positive for several types of islet cell autoantibodies, the patient was able to stop insulin therapy within three months of diagnosis.

## Case presentation

A 28-year-old Caucasian male presented to his general practitioner with a six-month history of feeling fatigued, polydipsia and polyuria, and weight loss of 25 kg. His initial weight was 128 kg (body mass index: 34.4 kg/m^2^). His general practitioner noted a raised capillary blood glucose level (17.9 mmol/L) and a raised capillary blood ketone level (6.3 mmol/L) and sent him to the emergency department. He had no other symptoms (i.e., no cough, dysuria, abdominal pain, diarrhea, fever, or shortness of breath). His medical history consisted of well-controlled asthma. He had no coexisting autoimmune conditions and no family history of type 1 or type 2 diabetes mellitus, however, his mother did have autoimmune hypothyroidism. At presentation, he was a non-smoker but drank alcohol occasionally.

On examination

He was conscious and alert, with a body temperature of 36.2°C, heart rate of 73 beats/min, respiratory rate of 18 breaths/min, blood pressure of 100/64 mmHg, and oxygen saturation of 97% on room air. Examination of his chest and abdomen demonstrated no abnormalities. Skin examination did not reveal any evidence of acanthosis nigricans. He weighed 103 kg with a height of 1.93 m (body mass index: 27.7 kg/m^2^) and a waist circumference of 94 cm (waist-to-height ratio less than 50%). He had no clinical features to suggest Cushing syndrome, acromegaly, or hyperthyroidism as a cause for secondary diabetes.

Investigations

Initial laboratory investigations revealed hyperglycemia, severe ketonemia, and metabolic acidosis, all indicating diabetic ketoacidosis (Table 1). His glycated hemoglobin (HbA1c) level was high, indicating that his blood glucose levels had been high for several weeks to months prior. His full blood count, serum C-reactive protein, renal function, liver function, and thyroid function tests were normal. A polymerase chain reaction (PCR) test for the coronavirus 2019 (COVID-19) infection was negative. His serum C-peptide level was in the low end of the normal range (relatively low for the level of hyperglycemia) and he had a high titer of glutamic acid decarboxylase autoantibodies (GADA), in keeping with a diagnosis of autoimmune (type 1) diabetes. A spot urine analysis demonstrated significant ketonuria (4+, 18 mmol/L) and significant glycosuria (4+, 110 mmol/L) during the admission (Table 1). An electrocardiograph demonstrated no abnormalities.

**Table 1:** Laboratory blood and urine test results on admission The presence of a blood glucose level more than 25 mmol/L, blood pH less than 7.35, positive serum and urine ketones, and increased anion gap metabolic acidosis all indicated diabetic ketoacidosis.

Blood test		Results	Laboratory reference ranges
Hemoglobin (g/L)		143	130-180
White cell count (10^9^/L)		4.8	4.0-11.0
Platelet count (10^9^/L)		194	150-400
Sodium (mmol/L)		135	133-146
Potassium (mmol/L)		4.1	3.5-5.3
Chloride (mmol/L)		96	95-108
Creatinine (µmol/L)		65	59-104
Glucose (mmol/L)		26.2	4.0-7.0
Urea (mmol/L)		3.4	2.5-7.8
Calculated serum osmolality (mmol/L)		299.6	275-295
Thyroid-stimulating hormone (mU/L)		2.08	0.3-4.2
Glycated hemoglobin (mmol/mol)		119	<42
C-reactive protein (mg/L)		2	<5
Amylase (U/L)		35	0-100
Total protein (g/L)		75	60-80
Albumin (g/L)		47	35-50
Alanine transferase (U/L)		38	10-60
Alkaline phosphatase (U/L)		116	30-130
Blood pH (venous blood gas analysis)		7.34	7.35-7.45
Bicarbonate (mmol/L)		21.7	23-30
Base excess		-3.7	-2 to +2
Anion gap (mmol/L)		19.3	4-16
Lactate (mmol/L)		1.71	0.2-1.8
Capillary ketones (mmol/L)		4.5	<0.4
C-peptide (nmol/L)		0.4	0.37-1.47
Glutamic acid decarboxylase autoantibodies (IU/mL)		238	<10
Urine test	Urine ketones (mmol/L)	18	<0.5
	Urine glucose (mmol/L)	110	<5.0

Treatment

The patient was commenced on intravenous insulin and fluids. He required only 0.01-0.03 units per kg body weight of intravenous insulin per hour for eight hours to get his blood glucose levels and ketone levels back to normal. He received a liter of intravenous sodium chloride infusion every eight hours over two days. After recovery, he went home on a twice-a-day regimen of biphasic insulin aspart (Novomix-30®) at a total dose of 0.35 units/kg body weight.

Outcome and follow-up

The patient was given a diagnosis of type 1 diabetes based on his clinical presentation and biochemical findings, whilst testing positive for several islet cell autoantibodies. One week after discharge from the hospital, a majority of his capillary blood glucose readings were 4.0-7.0 mmol/L with an increasing number of readings below 4.0 mmol/L. His total insulin dose was reduced to 0.31 units/kg. The patient admitted that he had put himself on a non-carbohydrate diet during the first-week post-discharge. A week later, his insulin regimen was changed to a basal-bolus regimen, however, he only required once daily insulin degludec (Tresiba®) at a dose of five units daily (0.049 units/kg body weight), indicating signs of partial clinical remission. A week later, the insulin dose was reduced to four units daily (0.039 units/kg). Another two weeks later, the insulin dose was reduced to three units daily (0.029 units/kg). A month later insulin therapy was stopped altogether. From the week after discharge up until the three-month point, he had been on a low-carbohydrate diet of 100-120 g/day to lose weight. Repeat blood tests at three months and six months post-discharge demonstrated glycated hemoglobin and C-peptide levels in the normal fasting range, indicating adequate pancreatic function (Table 2). Tests for the common islet cell autoantibodies (GADA, non-specific ICA, IA-2A, ZnT8A) were positive at the six-month point. Parietal cell antibodies, tissue transglutaminase antibodies, thyroid peroxidase antibodies, and adrenal antibodies were all negative: this ruled out pernicious anemia, celiac disease, autoimmune thyroid disease, and Addison’s disease, respectively. His vitamin D level was low, so he started taking a vitamin D supplement. At the nine-month point, his serum levels for the micronutrients (vitamin B12, folate, ferritin, magnesium, calcium, phosphate, selenium, zinc, vitamin D) were normal and his lipid profile was not adversely affected. His repeat glycated hemoglobin remained in the normal range, and C-peptide levels were still in the normal fasting range, indicating adequate pancreatic function (Table 2). Additionally, further tests for the islet cell autoantibodies (GADA, non-specific ICA, and IA-2A) were still positive at the nine-month point.

**Table 2:** Follow-up results after discharge from hospital Results indicated partial remission of type 1 diabetes in the presence of positive test for GAD autoantibodies at three months, and positive test for GAD, IA-2, ICA, and ZnT8 autoantibodies at six months and positive test for GAD, IA-2 and ICA autoantibodies at nine months post diagnosis. ICA: islet cell cytoplasmic autoantibodies; GAD: glutamic acid decarboxylase; IA-2: islet antigen-2; ZnT8: zinc transporter 8

Blood test	Results at 3 months	Results at 6 months	Results at 9 months	Laboratory reference ranges
Glycated hemoglobin (mmol/mol)	34	33	32	<42
Fasting glucose (mmol/L)	6.0	5.0	6.2	<7.0
C-peptide (nmol/L)	1.13	0.56	0.62	0.37-1.47
Glutamic acid decarboxylase autoantibodies (IU/mL)	125.4	233.7	1317.1	<10
Non-specific islet cell cytoplasmic autoantibodies	-	Positive	Positive	Negative
Islet antigen-2 autoantibodies (U/mL)	-	462.6	332.3	<10
Zinc transporter 8 autoantibodies (U/mL)	-	18	10	<15
Insulin autoantibodies (mg/L)	-	-	2	<5
Low-density lipoprotein cholesterol (mmol/L)	-	4.1	4.0	<3.5
High-density lipoprotein cholesterol (mmol/L)	-	1.2	1.2	>1.0
Triglyceride (mmol/L)	-	1.6	1.0	<2.0

The patient has maintained a body weight of 97 kg by combining regular exercise (weightlifting and cycling) with a low-carbohydrate diet of 50-70 g/day. His capillary blood glucose reading range was 4.5-7.0 mmol/L and capillary blood ketones were in the normal range. He will continue to self-monitor his capillary blood glucose and ketone levels, plus have three to six monthly biochemical monitoring in case he requires insulin therapy or any other anti-diabetic therapy in the future. The patient was provided with information concerning opportunities to be involved in ongoing research into prolonging beta cell survival in healthy individuals who test positive for islet cell autoantibodies (https://www.trialnet.org).

## Discussion

This study describes a case of a young adult who presented with clinical and biochemical features of type 1 diabetes with mild diabetic ketoacidosis. He was overweight but did not have any family history of type 2 diabetes mellitus. He required intravenous insulin and fluids on admission, and insulin therapy on discharge. He tested positive for several types of islet cell autoantibodies. He maintained a self-initiated low-carbohydrate diet. Despite the initial treatment with insulin, the patient’s insulin requirements declined rapidly until he was able to stop insulin therapy within three months following diagnosis. He has remained off insulin therapy or any antidiabetic medication, during which time his glycated hemoglobin levels have remained in the normal range.

The honeymoon phase of type 1 diabetes

The honeymoon (partial and complete remission) period in patients with type 1 diabetes has been extensively studied in the pediatric and adolescent population [4]. The pathogenesis of the honeymoon period remains unclear. There has been considerable debate over the clinical definition of the remission phase, and this has contributed to the varied prevalence rate (30-80%). Partial clinical remission has been defined as an insulin requirement of less than 0.5 units/kg of body weight per day and glycated hemoglobin (HbA1c) less than 7% (53 mmol/mol). Other studies have used an HbA1c level of 6% (42 mmol/mol) [2]. Another formula combining both values was proposed: the insulin dose-adjusted HbA1c (IDAA1c) defined as HbA1c (%) + (4 × {insulin dose in units/kg/24 h}), with a value of less than 9 indicating partial clinical remission. This formula considered the insulin dose and metabolic control and it correlated with C-peptide levels as a measure of residual beta cell function. It had also been validated by several large cohort studies and was recommended by the International Society for Pediatric and Adolescent Diabetes (ISPAD) [4,5].

A pediatric study demonstrated that 71 (69%) out of 103 children diagnosed with type 1 diabetes went into remission within a year (68 partial remissions, three complete remissions) [2]. The mean time to onset of partial remission was 28.6 days and the duration of partial remission was 7.2 months. The mean time to onset of complete remission was two months after diagnosis, and the duration of the complete remission was 3.7 months. The factors that influenced the onset of remission (honeymoon period) were older age group, absence of diabetic ketoacidosis at presentation, lower blood glucose levels, and higher blood pH at presentation, as compared with the patient in this case report. Longer duration of symptoms prior to presentation correlated negatively with both time of onset of the honeymoon period and its duration [2]. Another study in 15-34 years olds with antibody-positive type 1 diabetes revealed that normal body weight at diagnosis was an important factor for entering remission, whilst a low number of islet cell autoantibodies was important for the duration of remission [6]. In contrast, the patient in this case report was overweight at diagnosis and had a high number of islet cell autoantibodies. More studies are required to better predict the occurrence of partial clinical remission and duration of remission in adults with newly diagnosed type 1 diabetes.

There are a handful of case reports of prolonged honeymoon periods in adults with type 1 diabetes [7,8]. One study reported a 24-year-old Thai male who presented with a six-month history of polyuria, polydipsia, and weight loss, but no ketonemia. His Islet antigen-2 antibody (IA-A2) was positive, but GADA was negative initially. His initial insulin therapy was converted to oral anti-diabetic medication two months after diagnosis, and the oral medication was stopped two months later. He remained off insulin for five years after diagnosis [7]. Another study reported a 32-year-old African American female who presented with a three-week history of polyuria, polydipsia, and blurred vision discovered to have diabetic ketoacidosis and was diagnosed with type 1 diabetes (GADA-positive). Due to poor compliance and logistic issues, she stopped taking insulin four months after diagnosis and had normal glycated hemoglobin levels at 11 months and 14 months after diagnosis [8].

Although not true to definition, there are other case reports of patients being maintained on sitagliptin (dipeptidyl peptidase-4 inhibitor) during the remission phase. It was postulated that dipeptidyl peptidase-4 inhibitors can diminish daily insulin requirements and improve metabolic control without exacerbating the risk of hypoglycemia in patients with type 1 diabetes [9,10]. One such case is a 19-year-old South American male who presented with diabetic ketoacidosis and GADA-positive. He was treated with both insulin and sitagliptin (dipeptidyl peptidase-4 inhibitor). This patient was able to stop insulin therapy after eight weeks and continued the sitagliptin thereafter [9]. Another case involves a 67-year-old man who presented with diabetic ketoacidosis with both GADA and IA-2A positive. Three months after diagnosis, his insulin therapy was converted to sitagliptin [10].

Ketosis-prone type 2 diabetes

Ketosis-prone type 2 diabetes is an increasingly recognized phenomenon [11,12]. Affected patients are often non-Caucasian, male, middle-aged, overweight with other features of the metabolic syndrome (e.g., hypertension and dyslipidemia), and have a family history of type 2 diabetes. They present with new-onset severe hyperglycemia and ketosis or diabetic ketoacidosis and test negative for GADA and other islet cell autoantibodies. Physical examination commonly reveals abdominal obesity and acanthosis nigricans, indicating insulin resistance. The mechanism for this acute presentation remains unknown. However, studies have demonstrated that there is an acute inability of exogenous or endogenous glucose to stimulate beta cell insulin secretion combined with severe insulin resistance. Initial treatment involves intravenous insulin and fluid replacement. After weeks to months of subcutaneous insulin therapy, the majority are able to convert to diet alone or oral anti-diabetic medication [11,12].

Our patient was overweight and had a similar emergency presentation and treatment as described in patients with ketosis-prone type 2 diabetes. However, he did not have any family history of diabetes and had no other features of the metabolic syndrome. Additionally, he tested positive for several islet cell autoantibodies and his C-peptide levels have remained in the low-normal range, as opposed to the higher levels seen in patients with insulin resistance or type 2 diabetes.

Latent autoimmune diabetes in adults and in the young

Latent autoimmune diabetes, also called slow-onset type 1 diabetes or type 1.5 diabetes, is characterized by gradual autoimmune destruction of the pancreatic beta cells with a gradual decline in insulin production (GADA-positive) [13]. This can occur in adults (latent autoimmune diabetes in adults {LADA}) and in children (latent autoimmune diabetes in the young {LADY}). Patients present with polyuria, polydipsia, fatigue, visual changes, and weight loss. Diabetic ketoacidosis is rarely the first presentation of LADA. Diet-alone and/or oral anti-diabetic medication is the usual treatment regimen. Sulfonylureas are avoided because they speed up beta-cell fatigue. The speed of progression to requiring insulin in the future is variable and may be influenced by raised GADA levels [13,14]. The Immunology of Diabetes Society (IDS) has established three main criteria for diagnosing LADA. These include the adult age of onset (>30 years); the presence of any islet cell autoantibody; and the absence of insulin requirement for at least 6 months from diagnosis [15]. The first and third criteria are controversial. The use of insulin therapy on admission and for a short time thereafter is physician-dependent. Studies have demonstrated that early use of insulin may preserve beta-cell function as evidenced by a maintained stimulated C-peptide response, normal HbA1C levels, and a decrease in autoantibody concentrations [16]. Research into the optimum treatment for LADA and halting progression to type 1 diabetes are ongoing [16].

Our patient had insulin therapy at diagnosis and remained on a reducing dose for three months afterward. He was only two years below the age criteria for diagnosing LADA. It should be noted that our patient did have symptoms for up to six months prior to diagnosis, and one could speculate that he may not have developed ketonemia or required the initial insulin therapy if he had presented much earlier. Given the presence of several autoantibodies, he could have had latent autoimmune diabetes for several months or years prior and had an acute presentation due to some unknown short-lived exacerbating factor. All these clinical variables and uncertainties contribute to the controversies surrounding the definition and diagnostic criteria for latent autoimmune diabetes.

Predictive factors for partial remission of type 1 diabetes

Studies dedicated to predicting the honeymoon (partial remission) phase of type 1 diabetes in adults are insufficient. However, factors that may favor the onset of remission in children and adolescents include older age group, absence of diabetic ketoacidosis at presentation, lower blood glucose levels, higher blood pH, and normal body weight at presentation. Shorter duration of symptoms prior to presentation and low number of islet cell autoantibodies may favor a longer duration of remission [2,6]. On the contrary, our patient was overweight and had high blood glucose levels and diabetic ketoacidosis (albeit mild) at presentation. Our patient also had a long duration of symptoms and high number of islet cell autoantibodies that would favor a shorter duration of remission. Our patient demonstrated signs of partial remission within three weeks of diagnosis, adequate pancreatic function within three months of diagnosis, and continued to exhibit adequate pancreatic function without the need for insulin therapy, for over seven months whilst testing positive for several types of islet cell autoantibodies. Thus, the predictive factors derived from the pediatric and adolescent studies did not apply to our patient.

Low-carbohydrate diet and improving diabetic control

Our patient went on to a non-carbohydrate diet for the first week after discharge from hospital. He then increased this to a low-carbohydrate diet of 100-120 g/day for the next six months, before reducing his carbohydrate intake to a stable level of 50-70 g/day. He intended to get his weight down to healthy levels, but he had come across literature concerning the role of a low-carbohydrate diet in improving diabetic control. In recent times, patients have self-initiated low-carbohydrate diets with the hope of losing weight and improving diabetic control. However, low-carbohydrate diets have been criticized because of the risk of excessive intake of saturated fats and resultant dyslipidemia, inadequate intake of vitamins and micronutrients, poor energy intake, ketosis, and resultant cardiovascular risk [17]. Observational studies and small, randomized trials have suggested that low-carbohydrate diets might improve glycemic control, glycemic variability, and time spent in hypoglycemia in patients with type 1 diabetes [17]. However, low-carbohydrate diets cannot replace insulin therapy in patients with type 1 diabetes. Therefore, the fast-growing interest in low-carbohydrate dietary regimens amongst individuals with newly diagnosed type 1 diabetes requires an evidence-based management plan, which encompasses dietary, anthropometric, and biochemical monitoring.

Patient perspective

Though the patient exhibits adequate pancreatic function without the need for insulin therapy, he understands that this could still be a prolonged honeymoon period or latent autoimmune diabetes. Given that he has tested positive for several islet cell autoantibodies, the probability of becoming insulin-dependent at any time is very high. Stopping insulin therapy soon after an emergency admission requiring intravenous insulin and subsequent subcutaneous insulin therapy can be frightening for both patient and healthcare provider. Although the patient in this report is relieved that he is off insulin injections for time being, he is aware that the possibility of becoming insulin-dependent in the future still hangs in the air. The patient has kindly submitted his own account of events up until discharge from hospital (Appendix 1). 

## Conclusions

This is a case of partial clinical remission of diabetes in a young adult who presented with clinical and biochemical features of type 1 diabetes, tested positive for several islet cell autoantibodies, and maintained himself on a self-initiated low-carbohydrate diet. This patient is either going through a prolonged honeymoon phase of type 1 diabetes or has latent autoimmune diabetes. Sudden-onset symptomatic hyperglycemia requiring insulin therapy or other antidiabetic medication can arise at any time, therefore, support and regular biochemical monitoring is required.

We welcome continued research into improving beta cell survival and halting progression to insulin dependence during the honeymoon (partial remission phase) in patients with type 1 diabetes and in healthy individuals who test positive for islet cell autoantibodies.

## Appendices

Appendix 1: patient perspective

In the four years prior to my diagnosis, I was in a stressful situation having moved from a demanding and stressful academic situation straight into a physically and mentally tasking employment role. As a result, I began to suffer from periods of anxiety and possibly depression (although I never sought help or confirmation about either of these), which had a negative effect on my personal and private life. My efficiency at work started to deteriorate, and I found that the periods of anxiety started getting more prolonged and negatively affecting my quality of life. During this time, I was gaining weight due to a combination of poor diet (large amounts of processed sugar, fatty foods, and carbs), combined with little exercise. This was to sustain me during a period of exhaustion from working nearly 14 hours a day on average with little time to consider my basic needs. This diet continued throughout 2020, although the exercise stopped during the pandemic lockdown.

My partner started making comments about my health. In September 2020, my partner commented about how much ice-cold water I drank. I put this down to the summer heat. In the winter of 2020, my partner kept telling me that my breath had changed and the smell in the bathroom after I voided urine was unusual. My partner also noted that I was increasingly short-tempered with mood swings, advising me to seek professional help and take time off work. I was not receptive to this advice because of the immense pressure I was under at work.

By March 2021, I had been working from home late into the evening, seven days a week without time off for several months. I was unable to deliver on my increasing work commitments. At this point, the pressure to deliver on these commitments continued to increase with limited support.

In March 2021, I started having a very dry mouth regardless of how much water I drank. After a week, I stopped taking processed sugar altogether, as I suspected that this was diet-related and that my pancreas was reacting to the sugar. This did not relieve my symptom. I also found that I was voiding urine more often. I had gone from not voiding urine at night to voiding urine one to two times per night. My urine had become concentrated (syrup-like) with an unpleasant smell. My weight had begun to drop quickly. This continued until I was admitted to the hospital in August 2021.

By mid-July, I had lost four stones in weight and was feeling fatigued and exhausted. I was also losing my head hair. I believed the hair loss and weight loss were due to stress. My partner called our local general practitioner (GP) to express concerns about my health near the end of July 2021. I received a telephone consultation and was asked to come in for urgent blood tests. I later received a call from the GP informing me that I had diabetes and that the diabetes team would contact me the next day. I then received another call from the GP requesting that I attend the surgery that same day. I had some more blood work done, urine analysis, and a short physical examination done at the surgery. The GP then informed me that I needed to go to the local hospital emergency department immediately and should expect to be admitted. The GP gave me a handwritten letter and informed me that the emergency department had been informed and was expecting me.

Once admitted to hospital, I was given insulin and fluids. I remained on the fluids while being informed of my blood glucose and ketone levels after each test. Four days later, I went home with an insulin injection pen, which I initially used twice a day. At this point, I no longer had a craving for ice-cold drinks: something that I had been doing since the summer of the previous year.

I chose to reduce the amount of carbohydrates and sugars I was consuming and ensure that most of them were consumed during breakfast, and learn to better align the carbohydrates I was eating with the insulin I was taking. I found that this made it very easy to both identify my glucose response and keep the measurements within the limits I had set. Within the first few weeks, I found that I was regularly getting capillary glucose readings below 4.0 mmol/L, and had to rapidly reduce my insulin dose until I was taken off it completely. At this point, I began to reduce my weight by cycling and lifting weights. I also made it a point to reduce my workload and the anxiety that accompanied it.
